# Departure of Dr. Alfred Stille for Europe

**Published:** 1851-02

**Authors:** 


*Departure of Dr. Alfred Stille for Europe.*—We are pained to learn that Dr. Alfred Stille, of Philadelphia, who has been very zealous and active in promoting the lmeasures of reform in the medical profession, contemplated by the organizaiion of the American Medical Association, of which he is a member, has, on account of a serious cerebral attack, been compelled to abandon, for a time, the practice of his profession, and to seek in Europe a restoration of his health. May he reap the greatest benefit from his travels, and return in full vigor of mind and body.—*Charleston Med. Jovrnal.*

